# P.R.O.P.S. — A novel Pre-Operative Predictive Score for unresectability in patients with colorectal peritoneal metastases being considered for cytoreductive surgery (CRS) and hyperthermic intraperitoneal chemotherapy (HIPEC)

**DOI:** 10.1186/s12957-019-1673-x

**Published:** 2019-08-07

**Authors:** Zachary Zihui Yong, Grace Hwei Ching Tan, Nicholas Shannon, Claramae Chia, Melissa Ching Ching Teo

**Affiliations:** Division of Surgical Oncology, National Cancer Centre Singapore, 11 Hospital Drive, Singapore, 169610 Singapore

**Keywords:** Colorectal cancer, Peritoneal metastasis, CRS and HIPEC, Selection criteria, Unresectability

## Abstract

**Background:**

Twenty to thirty percent of planned cytoreductive surgery and hyperthermic intraperitoneal chemotherapy (CRS and HIPEC) procedures are abandoned intra-operatively. Pre-operative factors associated with unresectability identified previously were used to develop a Pre-Operative Predictive Score (PROPS), which was compared with current selection criteria—Peritoneal Surface Disease Severity Score (PSDSS), Verwaal’s Prognostic Score (PS) and Colorectal Peritoneal Metastases Prognostic Surgical Score (COMPASS), to determine which score provides the best prediction for unresectability.

**Methods:**

Fifty-six patients with peritoneal metastases of colorectal origin were included. Beta-coefficient values of significant variables (*p* < 0.05) were determined from multivariate analysis to develop PROPS. PROPS, PSDSS, PS and COMPASS were compared using a receiver operating characteristic curve to calculate its accuracy, sensitivity and specificity.

**Results:**

PROPS consisted of nine patient and tumour factors which were categorised into three groups: (i) poor tumour biology: previous inadequate resection, underwent multiple lines of chemotherapy and poorly differentiated or signet cell histology; (ii) heavy tumour burden: abdominal distension, palpable abdominal mass and computed tomography findings of ascites, small bowel disease and/or omental thickening; and (iii) active tumour proliferation: elevated tumour markers. Overall, PROPS achieved 86% accuracy with 100% sensitivity and 68% specificity, PSDSS achieved 85% accuracy with 100% sensitivity and 63% specificity, PS achieved 73% accuracy with 100% sensitivity and 68% specificity and COMPASS achieved 61% accuracy with 27% sensitivity and 100% specificity.

**Conclusions:**

PROPS is more effective in predicting unresectability as compared to PSDSS, PS and COMPASS, and has the added advantage of using solely pre-operative factors.

## 1.Introduction

Cytoreductive surgery (CRS) and hyperthermic intraperitoneal chemotherapy (HIPEC) have been showed to increase survival in patients with colorectal cancer peritoneal metastases (pCRC) [1, 2]. A proportion of CRS and HIPEC cases are abandoned intra-operatively due to extensive disease (i.e. high peritoneal carcinomatosis index (PCI) score) resulting in unnecessary laparotomy [3–5]. A way to circumvent this is to identify the unresectable cases pre-operatively. Pre-operative radiological investigations alone fail to accurately predict PCI score accurately [6]. One can also extrapolate the factors founds in prognostic scores such as Peritoneal Surface Disease Severity Score (PSDSS), Verwaal Prognostic Score (PS) and Colorectal Peritoneal Metastases Prognostic Surgical Score (COMPASS) for pre-operative selection outlined in Table 1 [7–9]. However, these prognostic scoring systems were established from studies that only included patients with pCRC that underwent successful CRS and HIPEC (i.e. completeness of cytoreduction score of 0 or 1), and were designed to select patients for complete resection or improved survival. In addition, both scores include intra-operative factors in their scoring model. Therefore, these scores are not primed to predict for unresectability pre-operatively. We have published an earlier study on preoperative factors associated with unresectability included all tumour types [10]. For this study, we only selected patients with pCRC. The aim was to develop a Pre-Operative Predictive Score (PROPS) for unresectability in patients with pCRC, and compare it with the PSDSS and PS, to determine which score best predicts for unresectability.

**Table 1:**
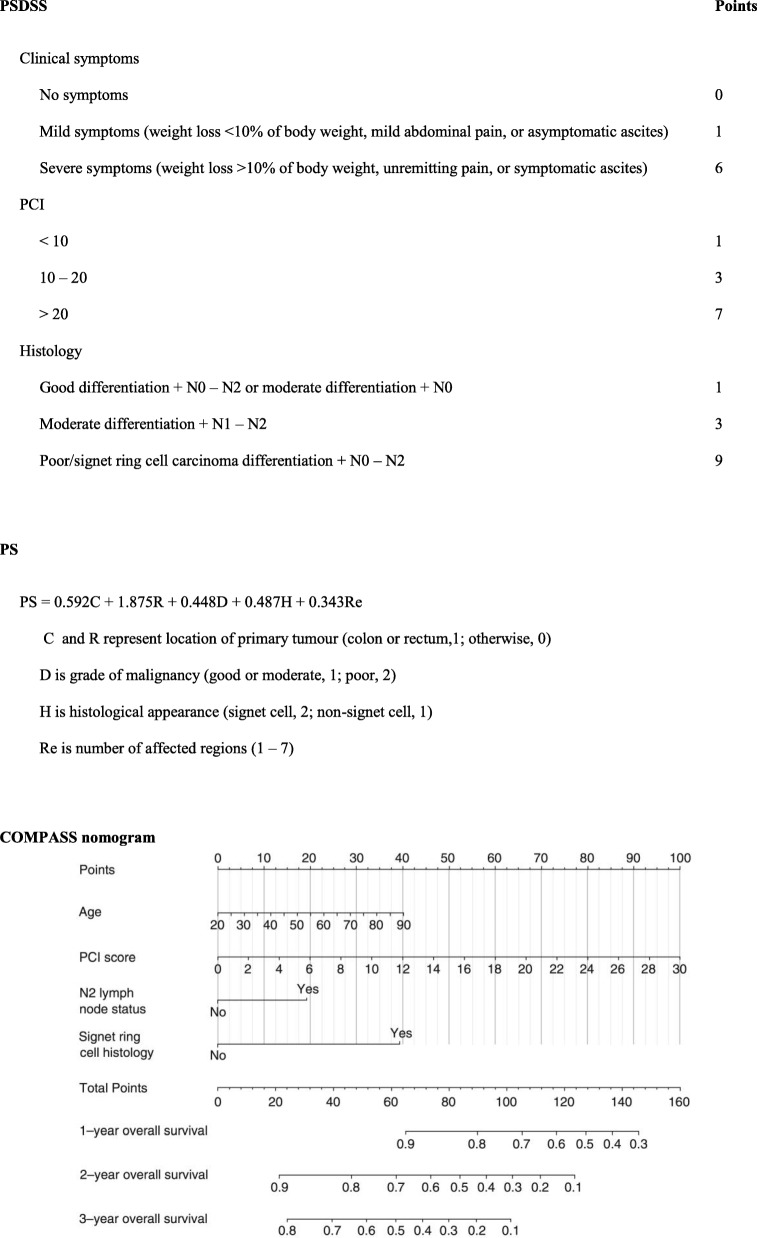
Variables in PSDSS, PS and COMPASS

## 2.Methods

This is a follow-up to a study that was conducted at the National Cancer Centre Singapore from April 2004 to May 2014 and was approved by the local Centralised Institutional Review Board. Data was retrospectively collected from a prospective CRS and HIPEC database of patients. Only patients with pCRC were included.

The patients included in this study had documented colorectal cancer with peritoneal metastases either on radiological imaging or during previous surgery. All cases were presented in a multidisciplinary meeting where surgical, medical and radiation oncologists, radiologist and pathologists were present, and the decision to proceed with CRS and HIPEC was determined after a consensus was reached. Clinical factors taken into consideration to formulate a decision include patients’ presentation, grade (e.g. presence of signet ring, mucinous or poorly differentiated cells) and stage of tumour, disease free-interval (DFI), response to previous therapies (e.g. chemotherapy or surgery) and radiological images. Patients recommended to undergo CRS and HIPEC were all without distant metastases as determined on imaging. In addition, they were of Eastern Cooperative Oncology Group (ECOG) status 0 or 1 [11].

Patients were grouped into two groups: the unresectable group was defined by inoperability determined intra-operatively, which resulted in the abandonment of planned CRS and HIPEC; the resected group was defined by completion of CRS and HIPEC regardless of the completeness of cytoreduction (CC) score, although it was noted that all patients who underwent CRS and HIPEC achieved either CC-0 or CC-1 [12]. The two groups were compared to identify pre-operative factors that will be useful to detect potentially unresectable patients which can help guide pre-operative decision-making and counselling and improve patient selection to decrease the chance of unnecessary exploration.

The clinical pre-operative factors that were analysed included the patients’ presentation, previous response to chemotherapy and/or surgical intervention as well as blood and radiological investigations. Tumour response to chemotherapy was evaluated with the Response Evaluation Criteria in Solid Tumours (RECIST) criteria [13]. PCI score was calculated intra-operatively during laparotomy. In addition, supplementary data was collected from clinical notes, electronic medical records and surgical records to complete both the PSDSS and PS for the same group of patients.

### 2.1.Statistical analysis

Between April 2004 and May 2014, 56 patients with pCRC were eligible for CRS and HIPEC. The first 31 patients (discovery set) were used to generate the model and the subsequent 25 patients were used to validate it (validation set). Using the discovery set, univariate analysis identified significant variables (*p* < 0.1) that were chosen for multivariate analysis. Weights attributed for the significant variables after multivariate analysis (*p* < 0.05) were obtained from the approximated beta-coefficient value (BC) from multivariate analysis to develop the scoring system (PROPS). Odd ratios (OR) and 95% confidence interval (95% CI) were also acquired from the multivariate analysis. The scoring systems of PROPS, PSDSS and PS were applied to the validation set to generate a receiver operating characteristic (ROC) curve to calculate its accuracy, sensitivity and specificity. The Youden’s index was used to identify the optimal cut-off value that gives maximum sensitivity and specificity based on the summary measurement of the ROC curve.

## 3.Results

Overall, the rate of unresectable cases was 13% (7/56). The demographics between the unresectable and successful groups were comparable except for PCI score and histology (Table 2). All unresectable cases were due to high PCI score with mean score of 24 (SD = 2.6). Of note, the unresectable cases had more tumours with signet ring cell and mucinous histology. Otherwise, the majority of the successful group achieved adequate cytoreduction (93% of CC-0 and CC-1).

**Table 2:** Demographics between unresectable and successful groups

Variables	Unresectable (*n* = 4)	Successful (*n* = 27)	*P* value
Age (mean)	49 (SD = 5.6)	49 (SD = 2.6)	0.97
Gender (female)	25% (1/4)	63% (17/27)	0.28
Race			0.21
Chinese	50%(2/4)	81.5% (22/27)	
Malay	0% (0/4)	0% (0/27)	
Indian	0% (0/4)	0% (0/27)	
Others	50% (2/4)	18.5% (5/27)	
ECOG			0.55
0	75% (3/4)	59% (16/27)	
1	25% (1/4)	41% (11/27)	
Overall comorbidity			
Cardiovascular	25% (1/4)	30% (8/27)	0.85
Pulmonary	100% (4/4)	11% (3/27)	0.78
Diabetes mellitus	0% (0/4)	19% (5/27)	0.35
Primary tumour location			0.50
Right-sided	75% (3/4)	44% (12/27)	
Left-sided	25% (!/4)	48% (13/27)	
Rectal	0% (0/4)	7% (2/27)	
Histology			0.00*
Signet ring cell	50% (2/4)	0% (0/27)	
Mucinous	50% (2/4)	26% (7/27)	
Adenocarcinoma			
Well differentiated	0% (0/4)	7% (2/27)	
Moderate differentiated	0% (0/4)	59% (16/27)	
Poor differentiated	0% (0/4)	7% (2/27)	
PCI score (mean)	24 (SD = 2.6)	10 (SD = 1.1)	0.00*
Completeness of cytoreduction			NA
CC-0	NA	41% (11/27)	
CC-1	NA	52% (14/27)	
CC-2	NA	7% (2/27)	

*Statistically significant results as *P* value is less than 0.05

Univariate analysis of the discovery set identified ten pre-operative factors significant for unresectability (Table 3). With regards to clinical presentation, patients in the unresectable group were more likely to complain of bloatedness (75% vs. 15%, *p* = 0.03), and were found to have palpable abdominal masses (25% vs. 0%, *p* = 0.04) on physical examination. In terms of disease factors, there were a greater proportion of high-grade tumours (50% vs. 4%, *p* = 0.01) in the unresectable group. For patients who had received treatment prior to the consideration of CRS and HIPEC, more patients from the unresectable group underwent multiple lines of chemotherapy (50% vs. 29%, *p* = 0.04), displayed progression of disease during chemotherapy (33% vs. 0.0%, *p* = 0.00) and/or had suboptimal initial resection (25% vs. 7%, *p* = 0.04). For pre-operative investigations, elevated tumour markers (100% vs. 50%, *p* = 0.03), as well as CT scan findings of ascites (100% vs. 9%, *p* = 0.00), omental thickening (100% vs. 4%, *p* = 0.00) and/or small bowel disease (25% vs. 8%, *p* = 0.01) were also more common in the unresectable group.

**Table 3:** Univariate analysis of preoperative factors associated with unresectability

	Unresectable (*n* = 4)	Successful (*n* = 27)	*P* value
Results	% (*n*)	% (*n*)	
Clinical presentation			
Bloatedness	75% (3/4)	15% (4/26)	0.03*
Altered bowel habits	50% (2/4)	12% (3/26)	0.12
Abdominal pain	50% (2/4)	15% (4/26)	0.17
Loss of weight	50% (2/4)	8% (2/26)	0.08
Nausea/vomiting	25% (1/4)	8% (2/26)	0.36
Abdominal distension	0.0% (0/4)	4% (1/26)	1.00
Abdominal mass	25% (1/4)	0.0% (0/26)	0.04*
Pouch of Douglas nodules	0.0% (0/4)	0.0% (0/26)	–
Disease factor			
High-grade tumour	50% (2/4)	4% (0/27)	0.01*
Prior neoadjuvant chemotherapy	75% (3/4)	96% (26/27)	0.25
Neoadjuvant cycles (median months)	9 (3 to 15)	6 (2 to 12)	0.14
Multiple lines of chemotherapy	50% (1/2)	29% (7/24)	0.04*
Response to chemotherapy			
Complete response	0.0% (0/3)	76% (16/21)	
Partial response	67% (2/3)	10% (2/21)	0.11
No response	0.0% (0/3)	14% (3/21)	0.20
Progressive disease	33% (1/3)	0.0% (0/21)	0.00*
Suboptimal resection	25% (1/4)	7% (2/27)	0.04*
Disease-free interval (median months)	14 (4 to 16)	16 (0 to 62)	0.23
Preoperative investigations			
Elevated tumour markers	100% (4/4)	50% (11/22)	0.03*
Thrombocytosis	50% (2/4)	15% (4/27)	0.16
Anaemia	25% (1/4)	41% (11/27)	1.00
Hypoalbuminemia	50% (2/4)	33% (9/27)	0.60
CT ascites	100% (4/4)	9% (2/23)	0.00*
CT omental thickening	100% (4/4)	4% (1/23)	0.00*
CT lymphadenopathy	25% (1/4)	17% (4/23)	1.00
CT small bowel disease	25% (1/4)	8% (2/23)	0.01*

*Statistically significant results as *P* value is less than 0.05

In addition to the above factors, those factors with *p* < 0.1 were chosen for multivariate analysis. All factors besides progression of disease during chemotherapy were still found to be significant. The remaining nine variables were categorised into three groups to generate PROPS (Table 4). Using the beta-coefficient value, individual scores were assigned to each variable in every group: (i) poor tumour biology (1 point each): suboptimal resection (BC 1.0, OR 0.17, 95% CI 0.05–0.63, *p* = 0.01), underwent multiple lines of chemotherapy (BC 1.0, OR 0.18, 95% 0.03–0.94, *p* = 0.07), and high-grade tumour (BC 1.0, OR 0.16, 95% CI 0.04–0.68, *p* = 0.03); (ii) heavy tumour burden (2 points each): sensation of bloatedness (BC 2.5, OR 0.16, 95% CI 0.04–0.58, *p* = 0.01), palpable abdominal mass (BC 2.5, OR 0.01, 95% CI 0.00–0.24, *p* = < 0.01) and computed tomography findings of ascites (BC 2.5, OR 0.02, 95% CI 0.00–0.33, *p* = < 0.01), small bowel disease (BC 2.5, OR 0.01, 95% CI 0.00–0.17, *p* = < 0.01) and omental thickening (BC 2.5, OR 0.14, 95% CI 0.02–0.78, *p* = 0.05); and (iii) active tumour proliferation (2 points): elevated tumour markers (BC 2.5, OR 0.10, 95% CI 0.01–0.84, *p* = 0.03).

**Table 4:** Development of PROPS with multivariate analysis

Variables	Beta-coefficient	Score	OR (95% CI)	*P* value
Poor tumour biology				
Suboptimal resection	1.0	1	0.17 (0.05–0.63)	0.01
Multiple lines of chemotherapy	1.0	1	0.18 (0.03–0.94)	0.07
High grade tumour	1.0	1	0.16 (0.04–0.68)	0.03
Heavy tumour burden				
Bloatedness	2.5	2	0.16 (0.04–0.58)	0.01
Palpable abdominal mass	2.5	2	0.01 (0.00–0.24)	< 0.01
CT ascites	2.5	2	0.02 (0.00–0.33)	< 0.01
CT omental thickening	2.5	2	0.01 (0.00–0.17)	< 0.01
CT small bowel disease	2.5	2	0.14 (0.02–0.78)	0.05
Active tumour proliferation				
Elevated tumour markers	2.0	2	0.10 (0.01–0.84)	0.03

**Fig. 1:**
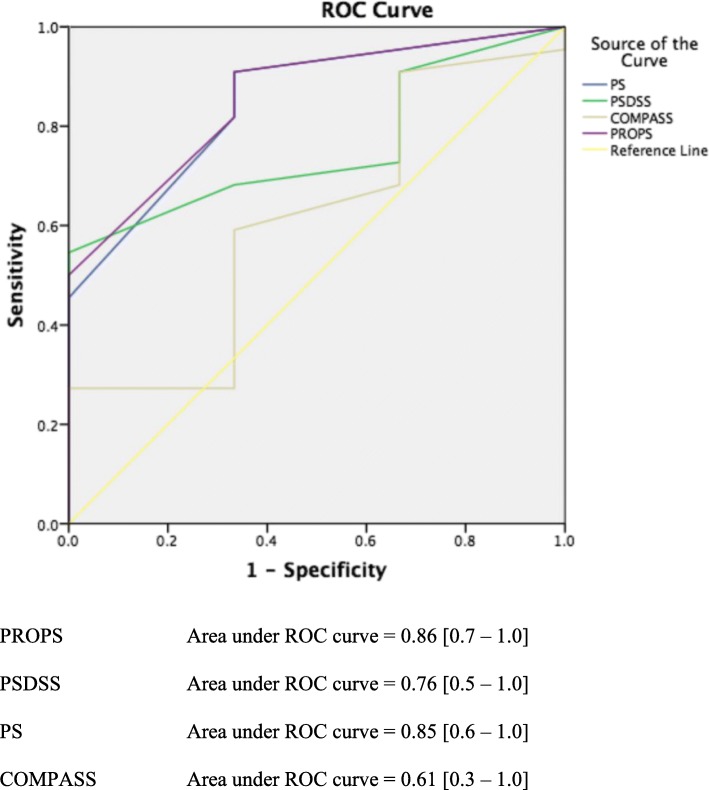
Receiver operating characteristic (ROC) curves for PROPS, PSDSS, PS and COMPASS. Area under the ROC curve for PROPS, PSDSS, PS and COMPASS when predicting unresectability are 0.86 (95% CI = 0.7–1.0), 0.76 (95% CI = 0.5–1.0), 0.85 (95% CI = 0.6–1.0) and 0.61 (95% CI = 0.3 = 1.0) respectively

Using the validation set for unresectability prediction (Table 5 and Fig. 1), PROPS achieved 86% accuracy with 100% sensitivity and 68% specificity at a cut-off of 3 (YI 0.68). PSDSS achieved 85% accuracy with 100% sensitivity and 63% specificity at a cut-off of 10 (YI 0.63). PS achieved 73% accuracy with 100% sensitivity and 68% specificity at a cut off of 3 (YI 0.68). And lastly, COMPASS achieved 61% accuracy with 27% sensitivity and 100% specificity at a cut off of 90 (YI 0.27). Of note, at a cut-off of 6, PROPS was able determine unresectability to near absolute certainty (specificity 95%).

**Table 5:** PROPS vs. PSDSS vs. PS vs. COMPASS

	Sensitivity	Specificity	Youden’s index
PROPS			
10	0.33	1	0.33
5.5	0.33	0.95	0.29
3.5	0.67	0.77	0.44
2.5	1.0	0.68	0.68
1.5	1.0	0.41	0.41
PSDSS			
17	0.33	0.90	0.24
14	0.67	0.86	0.53
11	0.67	0.81	0.48
9.5	1.0	0.63	0.63
8.0	1.0	0.59	0.59
PS			
3.25	0.00	0.77	−0.23
3.15	0.33	0.77	0.11
3.08	0.33	0.73	0.06
2.78	1.0	0.68	0.68
2.38	1.0	0.59	0.59
COMPASS			
81	0.27	0.67	−0.06
90	0.27	1.00	0.27
96	0.23	1.00	0.23
101	0.18	1.00	0.18
109	0.14	1.00	0.14

## 4.Discussion

Current pre-operative selection tools such as PSDSS, PS and COMPESS are useful in predicting survival outcomes, but may not be prime in identifying patients with unresectable disease as these studies excluded unresectable cases in their inclusion criteria. Moreover, these studies included intra-operative factors in their model (e.g. PCI in PSDSS and COMPASS and extent of carcinomatosis in PS) that preclude applicability in the pre-operative setting. The one other score available to predict unresectability in pCRC is the mCOREP [14]. We have considered incorporating mCOREP score into the study. However, as this is a retrospective study, we have a significant amount of missing data, in particular the CA125, which rendered the calculation of mCOREP score to be incomplete. The reason why CA125 was not part of our routine pre-operative was because it is specific for ovarian cancer instead of colorectal cancer. In a recent study conducted by Enbald et al., it was found that the mCOREP score (*p* = 0.9) and PSDSS (*p* = 0.09) are not predictive of opened and closed laparotomy. Instead, COMPASS score was found to have better predictive value for opened and closed laparotomy as compared to mCOREP score (*p* < 0.001) [14]. Additionally, Demey et al. have externally validated COMPASS to be superior over PSDSS in its prognostic ability. However, in our paper, we found COMPASS to perform the poorest in predicting unresectability compared to PROPS, PSDSS and PS. This may be attributed to the difference in our patient demographics as the patients in Demey et al. paper were considerably older (57 years old vs. 49 years old) and had lower PCI score (18 vs. 24), which result in discrepancy in the aggregation of COMPASS by approximately 25 points.

Our article demonstrated specific factors for the pre-operative identification of opened and closed cases that we condensed into a novel model—PROPS. At a cut-off of 3, we found that PROPS was best able to detect unresectability (specificity 68%) with the lowest rate of false positive (i.e. complete CRS/HIPEC, sensitivity 100%) compared to PSDSS and PS. At a cut-off of > 6, PROPS can distinguish unresectable cases close to absolute certainty (specificity 95%). The nine factors in PROPS were categorised into three main groups: (i) poor tumour biology, (ii) heavy tumour burden and (iii) active tumour proliferation.

### 4.1.Poor tumour biology

Advanced cancers have been reported to be one of the main reason for incomplete resection [15]. Extensive disease may preclude complete tumour resection because of technical difficulties or anatomical limitations. As a result, residual disease results in direct tumour extension, metastatic lymph nodes, microvascular invasion or tumour budding [15]. These retained tumours serve as a reservoir and cause larger spillage of tumour emboli, which subsequently result in a greater volume of peritoneal disease [16]. The same rationale holds for a previous suboptimal resection, which may be a harbinger of extensive peritoneal disease that is not remanable with CRS and HIPEC.

In a similar vein, high-grade tumours are also found to be associated with more advanced stage cancers [17–21]. Even in the absence of metastasis, high-grade tumours have a predilection to be locally invasive with increased risks of peritoneal seeding that may result in a hostile abdomen and frozen pelvis [16]. This may be due to their intrinsic properties of cell-cell adhesion disruption that promotes their aggressive behaviour with regard to invasion and metastasis [22]. With greater depth of invasion, it increases the propensity for peritoneal dissemination due to transcoelomic spread in high-grade tumours. Therefore, the degree of peritoneal involvement may be more substantial in high-grade tumours and that could translate to unresectability in CRS and HIPEC.

Acquired resistance to cancer therapies results in progressive disease and thus may require multiple lines of different chemotherapy for disease control. The correlation between progressive disease and multiple lines of chemotherapy may be the reason why the former was no longer found to be significant after multivariate analysis. The need for multiple lines of chemotherapy engenders underlying aggressive cancer [23]. In fact, Cottee et al. has recommended that progressive disease during systemic chemotherapy to be a contraindication to CRS and HIPEC in view of it being a poor prognostic marker to complete cytoreduction [24, 25]. In all, previous suboptimal resection, high-grade tumour and progressive disease whilst on chemotherapy forewarn biological aggressiveness of underlying tumour and may decrease the utility of CRS and HIPEC.

### 4.2.Heavy tumour burden

Symptomatic colorectal cancers, such as having the sensation of bloatedness, may be an indicator of the extent of the disease. This may also be the presenting symptom in patients who have massive ascites. It confers a poorer prognosis in terms of overall survival and disease-free survival [26, 27]. In addition, palpable abdominal masses may also suggest advanced disease [27]. In general, pCRC patients with clinical symptoms and signs tend to have larger cancers and more advanced local disease [28]. Larger tumours infiltrate the serosal surface over a larger surface area which may increase risk of tumour cells depositing onto the peritoneum via transcoelomic spread [29]. Therefore, having symptoms like bloatedness and detecting abdominal masses during examination are red flags for extensive pCRC that may decrease the chance of a successful CRS and HIPEC. Interestingly, abdominal distension alone was not found to be significant. This implies that the asymptomatic increase in abdominal girth alone is not as specific for unresectability than a symptomatic abdominal distension.

A cause of the aforementioned bloatedness may be due to ascites causing raised abdominal pressure. The formation of ascites is related to altered vascular permeability and obstructed lymphatic system due to the peritoneal disease [30]. This is also in keeping with our finding as high-grade tumours have also been reported to exhibit rapid disease progression that promotes accumulation of intra-peritoneal fluid [30]. As with the presence of clinical symptoms and signs, the formation of ascites is also a grave prognostic sign in pCRC [31, 32]. Studies have shown a positive correlation with the degree of ascites to the extent of tumour burden, that ascites formation occurred in late stages of tumour growth with heavier tumour burden [33]. In fact, there is a positive feedback loop that neoplastic spread in the peritoneal cavity promotes ascites formation which in turn favours deposition, fixation and growth of seeded malignant cells that result in greater volume of ascites [34, 35]. Therefore, CT finding of ascites is an indication of a high-volume peritoneal disease that may result in a higher risk for incomplete CRS and HIPEC.

Omental thickening is commonly seen in patients with pCRC. This is because the omentum is rich in lymphoid tissue and assists in the reabsorption of peritoneal fluid that facilitates neoplastic seeding. It has been shown that the presence of omental thickening connotes advanced disease [36]. On top of that, small bowels may also be inflicted with serosal tumour implants, frank bowel wall invasion and extensive adhesion formations in advanced pCRC as well [37]. This has provided us with evidence that the presence of omental thickening and small bowel disease on CT imaging may suggest underlying heavy tumour peritoneal disease, and thus result in a poorer chance of successful CRS and HIPEC. In addition, our study used CT as an imaging modality to evaluate pCRC pre-operatively. It has been shown that CT may not be sensitive in detecting early pCRC owning to the size of the tumour deposit [38–40]. This has resulted in many studies evaluating other modalities such as the MRI or PET scan to detect earlier and smaller pCRC [41–45]. Thus, CT-detected abnormalities may alone represent heavy volume disease due to its inherent inability in detecting early and small peritoneal deposits.

### 4.3.Active tumour proliferation

The overexpression of tumour markers signify active replicating of tumour cells [46]. Elevated tumour markers are a poor prognostic feature; with higher preoperative level, there will be a higher likelihood of extensive disease [47]. It has been shown that elevated tumour markers contribute to distortion of cellular architecture and facilitates tumour migration [48]. In experimental models, tumours that secrete tumour markers have a greater predilection for metastasis than non-secreting tumours [49]. In keeping with our results, we found that elevated tumour markers in the pre-operative setting were associated with a higher chance of unresectability. Interestingly, several studies have reported that the rise of tumour markers is greatest for liver metastases as compared to locoregional invasion like pCRC [50, 51]. One reason could be that these studies were limited to only carcinoembryonic antigen (CEA), while we included additional tumours markers such as CA 19-9. Besides CEA, we found raised CA 19-9 to be a poor prognostic factor. These findings concur with several studies that found association between raised CA 19-9 with pCRC [52–54]. Tumour cells that express CA 19-9 were found to be adherent to endothelial cells through E-selectin that promotes tumour metastasis [55, 56]. In particular, tumour cells in the peritoneal cavity bound to CA 19-9 monoclonal antibodies with a high frequency, which may explain the preponderance for peritoneal dissemination for CA 19-9 expressing tumours [57].

This study has a few limitations. Firstly, it is limited by its retrospective nature and single-centre design. The small number in the unresectable group also prohibits sub-group analysis and likely affects the statistical power of the analyses. In studies with small sample size, the chances of type 2 error (false negative) are technically higher without an effect on the rate of type 1 error (false positive). In general, as sample size increases, the chances of type 2 error decrease while the probability of type 1 error increases [58]. Therefore, we believe that even with a large sample size, the variables we found will remain significant. In addition, we are cognizant about the inherent problems of running multivariate analysis in small sample of data due to high standard errors. The challenge of accruing data for opened and closed laparotomy is that it is a hard to reach population. However, multivariate statistical models, specifically ordination (as with most of our variables used in our study), may be statistically powerful enough that the differences among samples are detected even at smaller sample size. That is, small sample size multivariate analysis may produce the same results as studies with large sample size studies. It is recommended that a minimum sample size of 58 individuals will suffice (our study is only off by two counts) [59]. Accordingly, we plan to conduct a larger scale prospective study on a separate group of pCRC patients, to further investigate the utility of PROPS and validate its utility.

## 5.Conclusion

The PROPS scoring system is a novel pre-operative scoring system that relies solely on pre-operative factors, and is as effective in predicting unresectability compared to the PSDSS, PS and COMPASS.

Moving forward, it will be important to perform external validation for PROPS, and to consider if PROPS can be applied to other tumour types besides colorectal to decrease the incidence of unresectability in planned CRS and HIPEC.

## Data Availability

The datasets generated during and/or analysed during the current study are available from the corresponding author on reasonable request.

## Abbreviations

BC: Beta-coefficient value
CC: Completeness of cytoreduction
CI: Confidence interval
COMPASS: Colorectal Peritoneal Metastases Prognostic Surgical Score
CRS: Cytoreductive surgery
DFI: Disease-free interval
ECOG: Eastern Cooperative Oncology Group
HIPEC: Hyperthermic intraperitoneal chemotherapy
OR: Odd ratios
PCI: Peritoneal carcinomatosis index
pCRC: Colorectal cancer peritoneal metastases
PROPS: Pre-Operative Predictive Score
PS: Verwaal Prognostic Score
PSDSS: Peritoneal Surface Disease Severity Score
RECIST: Response Evaluation Criteria in Solid Tumours
ROC: Receiver operating characteristic
SD: Standard deviation
YI: Youden’s index
